# Giant sixteen kilogram lymphangioma mesenteric cyst: An unusual presentation of a rare benign tumour

**DOI:** 10.1016/j.ijscr.2019.05.019

**Published:** 2019-05-14

**Authors:** Guy Aristide Bang, Paul Tolefac, Olivier Fola, Marcella Biyouma, Ulrich Bisay, Marc Leroy Guifo, Arthur Essomba

**Affiliations:** a Department of Surgery and Subspecialties, Faculty of Medicine and Biomedical Sciences, University of Yaoundé I, Yaoundé, Cameroon; b Surgical Unit, Yaoundé University Teaching Hospital, Yaoundé, Cameroon

**Keywords:** Mesenteric cyst, Transverse mesocolon, Giant abdominal mass, Low incomes country, Case report

## Abstract

**Background:**

Mesenteric cysts are rare abdominal benign tumours with an incidence of 1:100.000–250.000 surgical admissions located in the mesentery. Theirs presentations may range from incidental asymptomatic discovery during imaging to non-specific abdominal symptoms.

**Case presentation:**

We present the case of a 46 year old female who presented with 9 months history of progressive abdominal distension. CT scan showed a giant abdominal mass. After the necessary preoperative work up, a midline incision laparotomy was performed. Intraoperative findings were a mesenteric cyst originates from the transverse mesocolon. The cyst weighed 16 kg and histopathology analyses confirmed a lymphangioma mesenteric cyst.

**Discussion:**

In low incomes countries like our own, the interval between the onset of symptoms and consultation is often significant, leading to unusual and sometimes spectacular presentations at the time of diagnosis. To our knowledge, it is the heaviest mesenteric cyst reported in the literature to date.

**Conclusion:**

Mesenteric cysts may present as giant abdominal masses. The publication of this atypical case is a plea for us for the establishment of universal health coverage in our country in particular and in Africa in general.

## Highlights

• Mesenteric cysts may present as giant abdominal masses.
• It is the heaviest (16 kg) mesenteric cyst reported in the literature to date.
• In low incomes countries, the interval between the onset of symptoms and consultation is often significant, leading to unusual presentations.

## 1 Introduction

Mesenteric cyst is a generic term describing cysts occurring in the mesentery [[1], [2], [3], [4]]. It is a very rare benign abdominal tumour with an incidence of about 1:100.000–250.000 surgical admissions [2,5]. These benign tumours were first described in 1507 by Benevieni, a Florentine anatomist and in 1880, Tillaux performed the first successful mesenteric cyst resection. [2,6]. The aetiologies of these cysts are variable and largely unknown [6,7]. However, recent theories suggests that, These tumours usually arise from mesenteric lymphatic developmental abnormalities or from their traumatic rupture [2]. Mesenteric cysts are mostly located in the mesentery of the small bowel in 66% of the cases, mesentery of large intestine (ascending and transverse colon) in 33%. Less than 1% of the cases have been reported in the mesentery of the descending colon, sigmoid or rectum (around 1%) [2,6]. These cysts are more common in adults compared to children with the mean age of presentation in the fifth decade of life [7]. Mesenteric cysts are mostly asymptomatic and if present, symptoms are quite non-specific ranging from abdominal distension, bloating to dyspeptic symptoms [3]. While preoperative diagnosis is easily done by ultrasound, computed tomography scan (CT scan) or magnetic imaging resonance (MRI) [2], the definitive diagnosis is by histopathology.

Because mesenteric cysts are rare, very little information are available on them as most published studies consist of only a few cases. This has led to some false impressions and conclusions regarding these abdominal tumours [8]. The rarity of these conditions has also contributed to the fact that the correct preoperative diagnosis is infrequently made. Furthermore most mesenteric cysts described in literature are small in size and are managed by laparoscopy [2,4,6,7]. Herein, we report a case of a sixteen kilograms mesenteric cyst managed in a resource limited-setting in sub-Saharan Africa, at the Yaoundé’ University Teaching Hospital (Cameroon). This case report is in line with SCARE criteria [9].

## 2 Case presentation

A 46 year old female G0P0 was referred by a gastroenterologist to our surgical outpatient consultation for management of a giant intraabdominal mass. The history dates back nine months prior to her consultation, where she noted a progressive abdominal distension associated with constant non-colicky abdominal pains, nausea and dyspeptic symptoms. Due to limited financial resources and lacking health coverage, she does not consult in a hospital, but turns to traditional healers for several months.

**Fig. 1:**
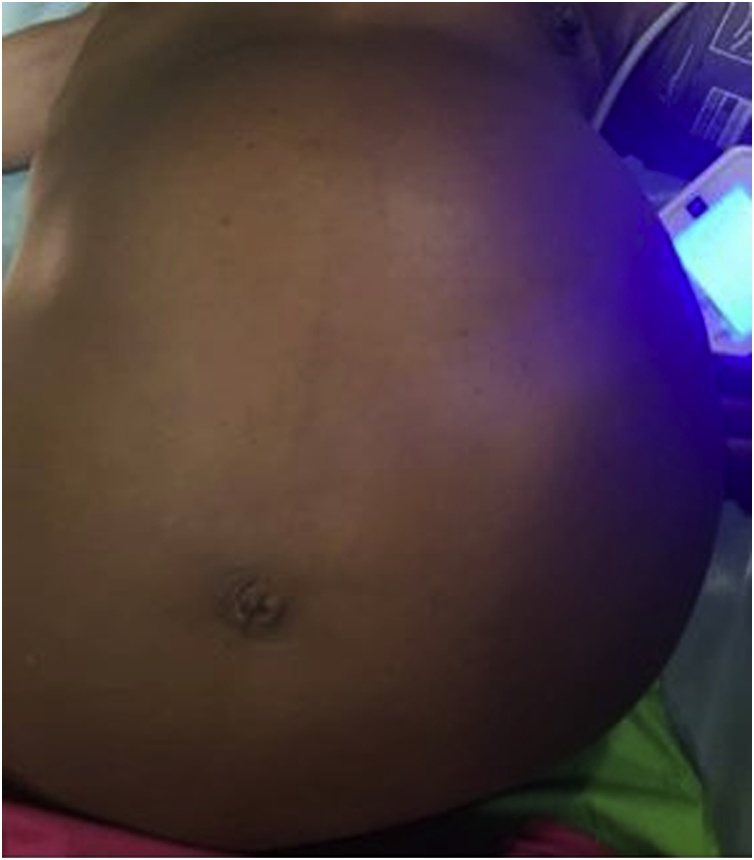
Distended abdomen before surgery.

The medical history was remarkable for a sudden acute flaccid lower limb paralysis since the age of 5 years, with an undetermined aetiology, having nailed her since then on a wheelchair. Physical examination showed a grossly distended abdomen (Fig. 1), with a large regular abdomino-pelvic mass lateralise to the left size measuring about 38 cm on it largest dimeter.

In front of this unusual case and having knowledge of the financial difficulties of the patient, our hospital decided to take charge of her free of charge.

**Fig. 2:**
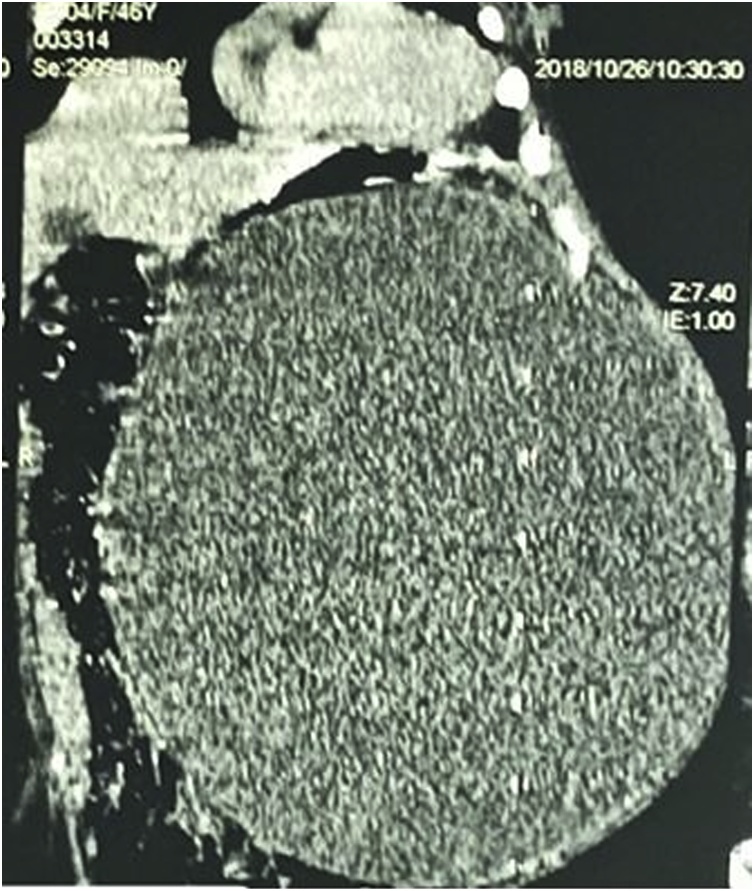
Abdominal CT scan showing a homogenous mesenteric abdomino-pelvic mesenteric cyst lateralise to the left, measuring 304 × 317 × 240mm.

**Fig. 3:**
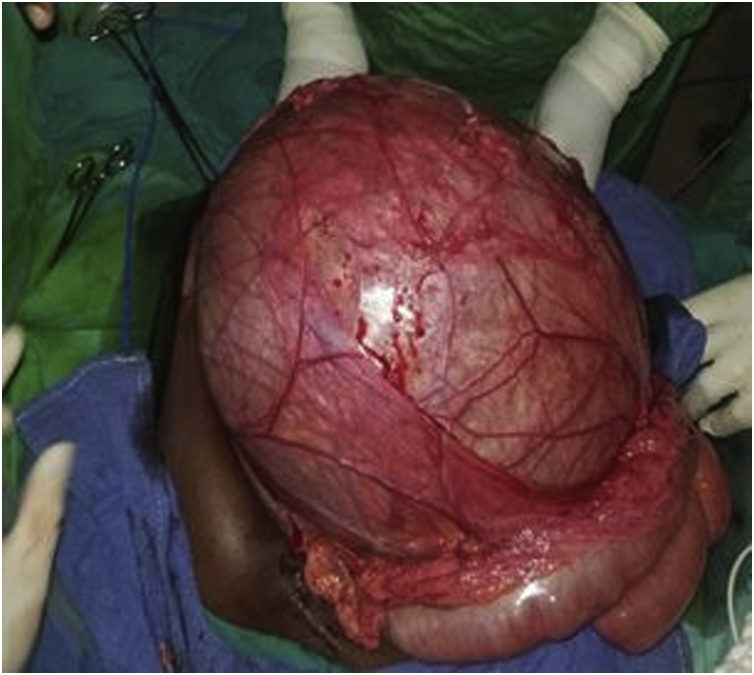
Intraoperative view of the mass, attached to the transverse mesocolon.

An abdominopelvic CT scan was then performed and showed an intraabdominal mesenteric cyst attached to the transverse colon (Fig. 2). She was then programmed for an exploratory laparotomy. Preoperative workups were normal. Under general anaesthesia, we performed a midline incision. Intraoperative findings consisted of a voluminous mesenteric mass attached to the transverse mesocolon between the posterior surface of the stomach and the transverse colon (Fig. 3). There was no ascites and no enlarged mesenteric lymph nodes. The mass was enucleated, with careful dissection of the transverse mesocolic vessels to which it was intimately bound.

**Fig. 4:**
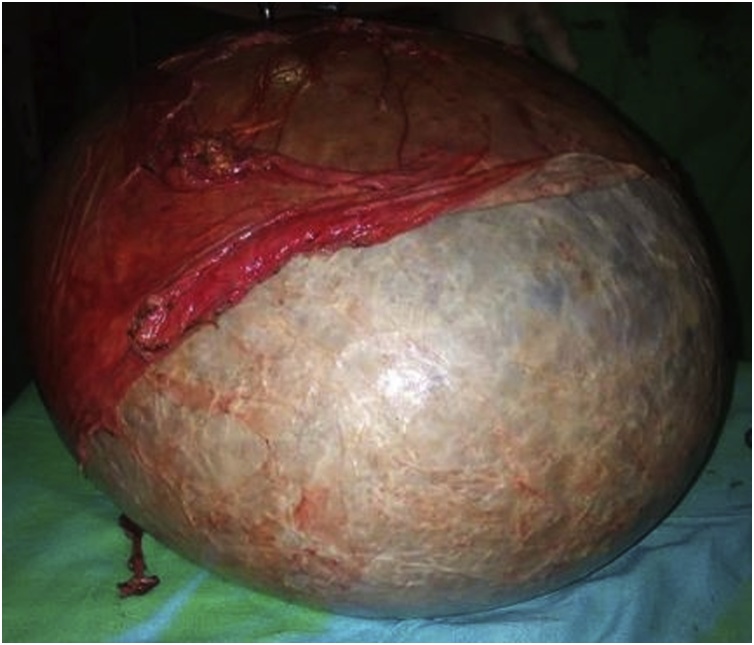
Gross macroscopic pathologic specimen of the removed cyst.

Grossly (Fig. 4), the mass measured about 33 × 30 x 25 cm. The mass was and weighed 16 kg. The postoperative course was uneventful with a discharge on postoperative day five. Histopathology revealed a lymphangioma of the transverse mesocolon.

## 3 Discussion

Mesenteric cysts are very rare intra-abdominal benign tumours that can pose serious diagnostic and therapeutic challenges [10]. Since the first reported case in 1507 [2], several cases have been described in literature. To the best of our knowledge this is the first report from Cameroon and the heaviest mesenteric cyst reported to date in the literature.

The presentation of a mesenteric cyst may range from incidental asymptomatic diagnosis during radiologic procedures to non-specific symptoms such as abdominal distension, abdominal pains and abdominal bloating. As a result to their non-specific symptomatology, there pose a great diagnostic challenge. Recent series suggests that the mean duration of symptoms at diagnosis is 2.6 months with a range of 12 h to 12 months [8]. In our case described, the patient presented after 9 months following onset of abdominal distension. Preoperative diagnosis is usually done with the use of imaging modalities such as ultrasonography, CT scan and or MRI [11,12]. While MRI is the most accurate investigation in diagnosing a cysts, ultrasounds and CT scan can readily distinguish between solid and cystic masses [13,14].

In 1880, Tillaux performed the first successful resection of a mesenteric cystic tumour and since then surgical resection has been the main stay of treatment [2]. This can be done by laparoscopy or by open surgery, with a favour on laparoscopy in recent reports [2,4,6,7].

In low-income African countries like ours, all of these "Western" treatment algorithms face an important challenge: health care financing. With a low health insurance coverage rate and often limited to the moneyed classes, the population is struggling to finance their health care. This leads to significant delays in consultation with unusual clinical presentations, but also increased morbidity and mortality. It is a sad daily reality for the physicians of these countries. In the past, unusual clinical presentation also due to a delay in consultation has been reported in our context [15]. The publication of this atypical case is a plea for us for the establishment of universal health coverage in our country in particular and in Africa in general.

In the current case, histopathology confirmed a giant lymphangioma cyst of the transverse mesocolon. This is in conformity of recent theories which suggests mesenteric cysts arise from developmental abnormalities of the mesenteric lymphatics or from their traumatic rupture [2].

Mesenteric cysts have a very low recurrence rate (0–13.6%) and patients have an excellent prognosis [11].

## 4 Conclusion

This case highlights the importance early clinical assessment and imaging in the management of patients with abdominal distension as practice in developed countries. However, limited resources common in low-income settings contribute to delayed consultations and then unusual presentations of diseases as shown in the present case: the heaviest mesenteric cyst reported to date.

## Funding sources

No Funding sources.

## Ethical approval

Our study is exempt from ethical approval by the ethics committee of the Faculty of Medicine and Biomedical Sciences of the University of Yaoundé I (Cameroon)

## Consent

Written informed consent was obtained from the patient for publication of this case report and accompanying images. A copy of the written consent is available for review by the Editor-in-Chief of this journal on request

## Author contribution

The patient was admitted and operated under the care of BANG Guy Aristide; he concepted the study. Paul TOLEFAC, Olivier FOLA and Marcella BIYOUMA collected data and wrote the paper. BANG Guy Aristide, BISAY Ulrich and marc le Roy GUIFO reviewed the paper. Arthur ESSOMBA gave the final approval.

## Registration of research studies

Our study isn’t a first-in-man One.

## Guarantor

Arthur ESSOMBA, Professor of general surgery at the Faculty of medicine and Biomedical Sciences of the university of Yaoundé I (Cameroon).

## Provenance and peer review

Not commissioned, externally peer-reviewed

## Conflict of interest

The authors declare no conflict of interest.
